# TNF-α-induced cardiomyocyte apoptosis contributes to cardiac dysfunction after coronary microembolization in mini-pigs

**DOI:** 10.1111/jcmm.12342

**Published:** 2014-07-31

**Authors:** Zhang-Wei Chen, Ju-Ying Qian, Jian-Ying Ma, Shu-Fu Chang, Hong Yun, Hang Jin, Ai-Jun Sun, Yun-Zeng Zou, Jun-Bo Ge

**Affiliations:** a Department of Cardiology, Shanghai Institute of Cardiovascular Diseases, Zhongshan Hospital, Fudan UniversityShanghai, China; b Department of Radiology, Shanghai Institute of Cardiovascular Diseases, Zhongshan Hospital, Fudan UniversityShanghai, China

**Keywords:** coronary microembolization, apoptosis, tumour necrosis factor-alpha

## Abstract

This experimental study was designed to clarify the relationship between cardiomyocyte apoptosis and tumour necrosis factor-alpha (TNF-α) expression, and confirm the effect of TNF-α on cardiac dysfunction after coronary microembolization (CME) in mini-pigs. Nineteen mini-pigs were divided into three groups: sham-operation group (*n* = 5), CME group (*n* = 7) and adalimumab pre-treatment group (*n* = 7; TNF-α antibody, 2 mg/kg intracoronary injection before CME). Magnetic resonance imaging (3.0-T) was performed at baseline, 6th hour and 1 week after procedure. Cardiomyocyte apoptosis was detected by cardiac-TUNEL staining, and caspase-3 and caspase-8 were detected by RT-PCR and immunohistochemistry. Furthermore, serum TNF-α, IL-6 and troponin T were analysed, while myocardial expressions of TNF-α and IL-6 were detected. Both TNF-α expression (serum level and myocardial expression) and average number of apoptotic cardiomyocyte nuclei were significantly increased in CME group compared with the sham-operation group. Six hours after CME, left ventricular end-systolic volume (LVESV) was increased and the left ventricular ejection fraction (LVEF) was decreased in CME group. Pre-treatment with adalimumab not only significantly improved LVEF after CME (6th hour: 54.9 ± 2.3% *versus* 50.4 ± 3.9%, *P* = 0.036; 1 week: 56.7 ± 4.2% *versus* 52.7 ± 2.9%, *P* = 0.041), but also suppressed cardiomyocyte apoptosis and the expression of caspase-3 and caspase-8. Meanwhile, the average number of apoptotic cardiomyocytes nuclei was inversely correlated with LVEF (*r* = −0.535, *P* = 0.022). TNF-α-induced cardiomyocyte apoptosis is likely involved in cardiac dysfunction after CME. TNF-α antibody therapy suppresses cardiomyocyte apoptosis and improves early cardiac function after CME.

## Introduction

Coronary microembolization (CME) is a common complication associated with the rupture of atherosclerotic plaques or microthrombi dropping in acute coronary syndromes or coronary interventions 1. Recent experimental studies have demonstrated that progressive myocardial contractile dysfunction caused 2,3 by CME develops in the absence of significant atherosclerotic obstruction in epicardial coronary artery. The immediate consequences of CME are a transient decrease in coronary blood flow with subsequent reactive hyperaemia and a moderate reduction in regional myocardial function that recovers partially within minutes 4. Such phenomenon of perfusion-contraction mismatch after CME, which is quite different from classical ischaemia heart disease (perfusion-contraction match), has been demonstrated in previous experimental study 5,6. The number of leucocytes infiltrating the area at risk was significantly greater in CME group, which implied that contractile dysfunction caused by CME was mediated by local inflammatory response characterized by leucocyte infiltration and TNF-α expression 7,8. Clinical studies have shown that serum TNF-α was increased significantly during iatrogenic trigger for plaque rupture 9. Skyschally *et al*. found that glucocorticoid treatment could prevent progressive contractile dysfunction by decreasing the infiltration of leucocytes 10, while our previous study confirmed this finding 11. To clarify the role of TNF-α in CME-induced cardiac dysfunction, Skyschally *et al*. and his colleagues 12 pre-treated the CME models with TNF-α antibody 30 min. before CME. They found that this treatment attenuated the progressive cardiac dysfunction caused by CME. Importantly, data from other studies have supported that TNF-α was involved in the deterioration of heart function after CME as well 7,8,13. However, the downstream signal pathways of TNF-induced heart dysfunction after CME are still unclear.

As we know, TNF-α is one of most critical inflammatory cytokines involved in the pathogenesis and progression of heart failure, atherosclerosis, plaque rupture and restenosis after stenting and myocardial ischaemia/reperfusion injury 9,14,15. It is secreted by a number of cell types, including monocytes/macrophages 16, fibroblasts 17 and cardiomyocytes 18,19. TNF-α exerts its effects by binding to its receptors (TNFR1 and TNFR2) and regulating multiple cellular processes, including oxidative stress 20, nitric oxide synthase expression 21,22, activation of NF-κB 23, apoptosis 24 and so on. While pathways of oxidative stress, sphingosine production 8 and NF-κB activation 25 have been well characterized after CME, it was still unclear about the association between TNF-α expression and the occurrence of cardiac apoptosis in CME, although a few experimental studies indicated that apoptosis biomarker was increased after CME (in rats) 26 and the occurrence of apoptosis 27. However, it was unclear for the direct causal relation between TNF-α expression and cardiac apoptosis.

Following binding of TNF-α to TNFR1, silencer of death domain dissociates from TNFR1 complex, and TNFR1-associated death domain is recruited to the receptor. This allows for the initiation of apoptosis in vascular endothelial cells and cardiomyocytes 24. This process involves both recruitment of caspase-8 (extrinsic pathway) and activation of caspase-3 28. It was unknown whether TNF-α-induced cardiac apoptosis was involved in the protection of TNF-α antagonist in CME.

Our objective of this study focused on the occurrence of cardiac apoptosis after CME, and tried to explore direct relation between the effect of TNF-α and cardiac apoptosis. We also wanted to clarify whether reduction in cardiac apoptosis was involved in the cardiac protection of TNF-α antibody in CME.

## Material and methods

### Animal preparation and experimental protocol

Nineteen mini-pigs (22–26 kg) [SCXK (Shanghai) 2007-0013] of either sex were divided into three groups. Group one: Sham-operation group (*n* = 5); Group two: CME group (*n* = 7) and Group three: TNF-α antibody pre-treatment group (*n* = 7)—intracoronary received 2 mg/kg adalimumab (TNF-α antibody; Abbott, Queenborough, UK) 30 min. before CME. Animals were initially sedated *via* intramuscular injection of combination ketamine (20 mg/kg) and diazepam (2 mg/kg). Anaesthesia was maintained with intravenous 3% pentobarbital sodium. Vascular sheaths (one 7F and one 6F) were placed in the right femoral artery and vein respectively.

The experimental investigation conformed to the Guide for the Care and Use of Laboratory Animals published by the US National Institutes of Health (NIH Publication No. 85-23, revised 1996), and the protocols were approved by the Animal Care and Use Committee of Fudan University, China (A5732-01).

### Coronary microembolization

Detailed experimental steps to generate the mini-pig CME model have been previously described 11. White-stained polystyrene microspheres with a diameter of 42 μm (Dynospheres; Dyno Particles; Lillestrøm, Norway) and a mean dosage of 120,000 was selectively infused into left anterior descending artery within 30 min. Saline was injected instead of microsphere in the sham-operation group, while TNF-α antibody was injected before CME in the treatment group. Systemic haemodynamics was monitored before the procedure, and at 2nd and 6th hour, and 1 week after CME.

### Post-mortem analysis

One week after the procedures, hearts were excised and sectioned into five slices from apex to base in a plane parallel to the atrioventricular groove. We selected the second and third slices approached to apex for histological detection. Approximately, 200–300 mg of transmural myocardium, from the left ventricle anterior walls, was immediately frozen in liquid nitrogen and stored at −70°C for RT-PCR and Western blot. The formaldehyde-fixed specimens were embedded in paraffin and sectioned into slices of 5 μm thickness for haematoxylin and eosin staining, immunohistochemical analysis and terminal deoxynucleotidyl transferase-mediated dUTP nick end-labelling (TUNEL) staining. Other myocardium was immersed in a 0.2 mol/l sodium phosphate buffer (pH 7.4) containing 1.0% nitroblue tetrazolium chloride (NBT; Sigma-Aldrich, Deisenhofen, Germany) for 20 min. at 37°C to demonstrate the presence of myocardial necrosis after operation.

Haematoxylin and eosin staining was also applied to demonstrate the presence of myocardial microinfarcts. The area of necrosis was calculated from 10 random fields (×200) of each slice using Leica DFC 320 digital software (Leica Microsystems Imaging Solutions Ltd, Cambridge, UK), and the percentage of each slice was calculated and averaged. The observers who performed histological analyses were blinded to the results of study grouping.

### Magnetic resonance imaging

Magnetic resonance imaging (MRI) was performed at baseline, 6th hour and 1 week after operation using 3.0-T Siemens scanner (MAGNETOM Verio; Siemens AG Healthcare, Erlangen, Germany) with a maximum slew rate of 200 mT/m/msec. and maximum gradient strength of 40 mT/m. Transverse, two-chamber and four-chamber LV long-axis scout images were obtained to determine the final short-axis image plane. Cine MRI was acquired in contiguous short-axis planes from the apex to the base of the heart to measure LV function. After the cine was obtained, mini-pigs received an intravenous bolus of 0.05 mmol/kg gadolinium DTPA (Gd-DTPA; Magnevist; Schering, Berlin, Germany) at a rate of 4 ml/sec. by means of an infusion pump followed by a 10 ml flush of saline at a rate of 4 ml/sec. Electrocardiograph was recorded simultaneously, and MRI detection included the evaluation of left ventricular ejection fraction (LVEF), left ventricular end-systolic volume (LVESV) and left ventricular end-diastolic volume (LVEDV). Two observers performed MRI analysis were blinded to the results of grouping.

### Measurement of serum levels of TNF-α, IL-6 and Troponin T

Serum samples were obtained at baseline, 2nd hour, 6th hour and 1 week after CME or sham-operation, and stored at −70°C for detection. Serum concentrations of TNF-α and IL-6 were determined by ELISA assay (Catalog no: PTA00 and P6000; R&D company, Minneapolis, MN, USA). Serum level troponin T was analysed by immunoturbidimetry (Hitachi 7600-020 automatic biochemistry analyzer).

### TUNEL

Myocardial apoptosis was detected by TUNEL staining using a commercially available kit (In Situ Apoptosis Detection Kit, catalog number: TA5353; R&D Company), which was supplemented with a cation that specifically enhanced the labelling of apoptotic cardiac cells. In each specimen, paraffin slices were incubated with TUNEL labelling mixture and counterstained with nuclear fast red. TUNEL-positive cardiomyocyte nuclei, which were blue in colour, were counted in 10 random high-power fields (HPFs, ×400), and the average number of TUNEL-positive cell nuclei in each HPF was calculated by a Leica DFC 320 digital camera (Leica Microsystems Imaging Solutions Ltd).

### RT-PCR analysis

Total RNA was extracted from the anterior wall of myocardium using trizol (Invitrogen, Carlsbad, CA, USA), and dissolved in distilled water. RNA concentration was determined by measurement of optical density at 260 nm. Reverse transcription was performed with 1 μg of total RNA and oligo(dT) primers in a 20-μl reaction according to the manufacturer's protocol (PE Applied Biosystems, Waltham, MA, USA). The sequences of the 5′-sense primers and the 3′-anti-sense primers used in this study were shown in Table1. The amplified products were electrophoresed on a 1.5% agarose gel and stained with ethidium bromide. Resulting CT values were evaluated and normalized to GAPDH. These values were then averaged for each duplicate.

**Table 1:** The sequences of the sense primers and anti-sense primers used in this study

mRNA	Sense primer	Anti-sense primer	Products size
TNF-α	5′-CGAACAGGCAGCCGGACGAC-3′	5′-CAAGGGGCCAGCTGGAAACTCTT-3′	180 bp
P53	5′-CCCCAGCATCTCATCCG-3′	5′-CAAACACGCACCTCAAAGC-3′	254 bp
Caspase-3	5′-TAATTCAGGCCTGCCGAGGCACA-3′	5′-TCAGCGCTGCACAAAGTGACTGG-3′	330 bp
Caspase-8	5′-CTGCCTACAGGGTCA-3′	5′-TCCGTGCTACACTAAAA-3′	471 bp
GAPDH	5′-TCATCAGCAATGCCTCCTGTACCA-3′	5′-TATTTGGCAGGTTTCTCCAGACGG-3′	328 bp

TNF-α: Tumour necrosis factor-alpha.

### Immunohistochemistry and Western blot

All of 19 mini-pigs' heart were subjected to immunohistochemistry and Western blot analyses. Paraffin-embedded heart specimens were cut into 5-μm sections, deparaffinized in xylene, and dehydrated in graded alcohol. Endogenous peroxidase activity was quenched with 3% hydrogen peroxide. After rinsing with PBS, slices of each myocardium were incubated overnight at 4°C with mouse monoclonal anti-porcine TNF-α antibody (1:100, Catalog no: MAB6903, R&D Company), rabbit polyclonal anti-porcine IL-6 antibody (1:400, Catalog no: ab6672, Abcam, Cambridge, MA, USA), rabbit polyclonal anti-porcine caspase-3 antibody (1:400, Catalog no: ab4051; Abcam) and goat polyclonal anti-porcine caspase-8 antibody (1:200, Catalog no: sc-6136; Santa Cruz, Dallas, TX, USA) for immunohistochemistry. According to the protocol of Superpicture Polymer Detection Kit (Catalog no: 87-9663, Zymed laboratory, Invitrogen), HRP-polymer conjugate was added and incubated for 10 min. After a wash step, the DAB chromogen was added. Slices were counterstained with haematoxylin and examined by light microscopy. Positive-staining area per cent was calculated in 10 random high-power fields (HPFs, ×400) by a Leica DFC 320 digital camera (Leica Microsystems Imaging Solutions Ltd).

Detail experimental steps of the Western blot procedure have been previously described 11. Protein extracts from anterior heart wall were prepared, and of that 30-μg total proteins was separated by SDS-PAGE and transferred to polyvinylidene difluoride (PVDF) membranes. Membranes were then incubated with mouse monoclonal anti-porcine TNF-α antibody (1:100, Catalog no: MAB6903; R&D Company) or rabbit polyclonal anti-porcine IL-6 antibody (1:200, Catalog no: ab6672; Abcam) overnight at 4°C. The membrane was then washed three times for 5 min. each in PBS, followed by incubation with horseradish peroxidase-conjugated anti-rabbit IgG secondary antibody (1:5000) for 2 hrs. Membranes were then washed three times for 5 min. in PBS. The membranes were developed using enhanced chemiluminescence reagent (ECL). Finally, band densities of TNF-α and IL-6 were detected.

### Total collagen analysis

The extent of fibrosis was detected by Masson Trichrome stain. It was performed to distinguish areas of collagen, which was shown in blue colour. The percentage of blue staining was examined by light microscopy, and analysed by a Leica DFC 320 digital camera (Leica Microsystems Imaging Solutions Ltd). Collagen volume fraction (CVF) was calculated by averaging the percentage area of stained tissue within 10 fields (×200) randomly selected from the infarct area on each slice.

### Statistical analysis

Data were expressed as mean ± SD. Data from MRI (including LVESV, LVEDV and LVEF) and serum levels of TNF-α, IL-6 and troponin T at each time-point were estimated by two-way anova analysis, while one-way anova was performed for analysis of apoptosis (average apoptosis nuclei), RT-PCR (CT values), Western blot (average band density), positive-staining area per cent in immunohistochemistry and CVF among three groups. Student's *t*-test was performed for necrosis-area analysis between CME and antibody pre-treatment group. Correlation analysis (Spearman test) was performed to evaluate the correlations among cardiac volume, LVEF and average number of apoptosis nuclei. Statistical analyses were performed with SPSS software (Version 18.0). All *P*-values were two-sided, and *P* < 0.05 was considered statistically significant.

## Results

### Systemic haemodynamic and coronary angiogram

There were no significant changes in heart rate, blood pressure or coronary flow evaluated by Thrombolysis in Myocardial Infarction before and after the procedure (Table2).

**Table 2:** Changes of heart rate, blood pressure and TIMI flow grade before and after procedure

	Sham-operation (*n* = 5)		CME (*n* = 7)		TNF-α antibody +CME (*n* = 7)	
	Pre-	Post-	Pre-	Post-	Pre-	Post-
Heart rate (bpm)	84.4 ± 9.5	92.6 ± 8.6	89.2 ± 6.4	85.8 ± 10.2	92.0 ± 8.2	88.4 ± 7.9
SBP (mmHg)	94.2 ± 5.8	90.0 ± 4.5	94.7 ± 5.2	89.3 ± 7.3	88.5 ± 7.2	91.6 ± 3.6
DBP (mmHg)	65.1 ± 5.1	62.3 ± 7.0	60.8 ± 6.6	62.6 ± 4.3	64.4 ± 5.9	68.0 ± 4.3
TIMI grade	III	III	III	III	III	III

Pre-: pre-procedure; Post-: post-procedure; CME: coronary microembolization group; DBP: diastolic blood pressure; SBP: systolic blood pressure; TIMI: Thrombolysis in Myocardial Infarction (a method for grading the coronary flow).

### Post-mortem analysis

**Figure 1:**
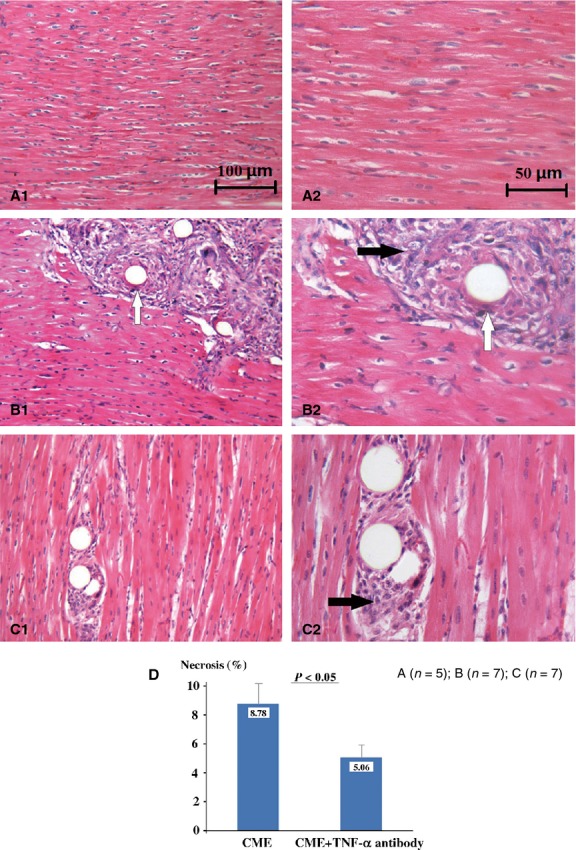
Anterior myocardium of three groups with haematoxylin and eosin staining. (**A**) Sham-operation; (**B**) CME group; (**C**) treatment group; ‘1′=×200; ‘2’=×400. (**D**) Comparison of the average area of microinfarctions between group B and group C. White arrows: pointed to microsphere; black arrows: pointed to microinfarction.

NBT and haematoxylin and eosin staining were performed. We detected obvious microinfarcts and polystyrene microspheres in the anterior wall of the CME group (microembolization-associated myocardium), which were not observed in sham-operation group. Leucocyte infiltration in the embolized myocardium was also increased in the CME group. Adalimumab (TNF-α antibody) not only greatly prevented leucocyte infiltration, but also decreased total areas of necrosis and microinfarcts in treatment group (Fig.1).

### MRI (3.0 T)

MRI using 3.0-T Siemens scanner was performed at baseline, 6th hour and 1 week after operation. Changes in cardiac volume and function at different time-points were shown in Table3. There was no significant difference at any time-point in sham-operation group. However, in the CME group, LVESV was significantly increased at 6th hour, and remained elevated for at least 1 week after CME. In contrast, LVEF was decreased. We found that pre-treatment with TNF-α antibody significantly improved cardiac function (LVEF-6th hour: 54.9 ± 2.3% *versus* 50.4 ± 3.9%, *P* = 0.036; LVEF-1 week: 56.7 ± 4.2% *versus* 52.7 ± 2.9%, *P* = 0.041).

**Table 3:** Cardiac dimension and function of Three Groups detected by Magnetic resonance imaging at different time-points

	Sham-operation group (*n* = 5)			CME group (*n* = 7)			TNF-α antibody+CME (*n* = 7)		
	Base	6H	1W	Base	6H	1W	Base	6H	1W
LVESV (ml)	16.8 ± 2.2	17.2 ± 1.6	16.9 ± 1.4	17.5 ± 3.7	23.0 ± 2.6*‡	21.1 ± 2.4*‡	17.0 ± 2.4	20.1 ± 2.8*	19.5 ± 2.3
LVEDV (ml)	41.2 ± 3.0	40.7 ± 2.6	42.1 ± 2.0	42.7 ± 2.3	46.4 ± 3.5	44.4 ± 2.6	42.0 ± 2.2	44.4 ± 4.5	44.9 ± 1.9*
LVEF (%)	58.6 ± 2.0	56.8 ± 2.6	58.9 ± 2.4	57.6 ± 3.4	50.4 ± 3.9*‡	52.7 ± 2.9*‡	58.7 ± 2.1	54.9 ± 2.3§	56.7 ± 4.2§

* Compared with BASE, *P* < 0.05.

‡ Compared with sham-operation group, *P* < 0.05.

§ Compared with CME group, *P* < 0.05.

Compared with 6H, *P* < 0.05.

6H: six hours after operation; 1W: 1 week after operation; LVEDV: left ventricular end-diastolic volume; LVEF: left ventricular ejection fraction; LVESV: left ventricular end-systolic volume.

### Expression of serum TNF-α, IL-6 and Troponin T

Compared with concentration at baseline (122.8 ± 28.8 pg/ml), the serum level of TNF-α in the CME group was increased significantly at 2nd hour after CME (297.4 ± 38.4 pg/ml, *P* < 0.01); this effect was maintained up to 6 hrs after CME (242.6 ± 33.8 pg/ml, *P* < 0.01). One week later, the TNF-α level was decreased to basal level (130.8 ± 30.7 pg/ml, *P* < 0.01). In the TNF-α antibody pre-treatment group, serum levels of TNF-α were slightly decreased. However, the decrease was not statistically significant (2nd hour: 264.3 ± 41.2 *versus* 297.4 ± 38.4 pg/ml, *P* = 0.213; 6th hour: 213.4 ± 26.8 *versus* 242.6 ± 33.8 pg/ml, *P* = 0.261; Table4). Serum levels of IL-6 were not significantly altered by TNF-α antibody treatment. Troponin T, a classic cardiac injury biomarker, was also evaluated at different time-points after CME, shown in Table4. Compared with sham-operation group, it was increased significantly in CME group. TNF-α antibody treatment decreased troponin T level after CME.

**Table 4:** Three Groups' Serum levels of TNF-α, IL-6 and troponin T at different time-points

	Sham-operation group (*n* = 5)				CME group (*n* = 7)				Antibody treatment group (*n* = 7)			
	Base	2H	6H	1W	Base	2H	6H	1W	Base	2H	6H	1W
TNF-α (pg/ml)	115.4 ± 30.2	127.9 ± 29.8	130.4 ± 24.5	108.0 ± 27.4	122.8 ± 28.8	297.4 ± 38.4*,§	242.6 ± 33.8*,§	130.8 ± 30.7†,‡	130.5 ± 31.6	264.3 ± 41.2*,§	213.4 ± 26.8*,†,§	103.7 ± 32.8†,‡
IL-6 (pg/ml)	43.06 ± 24.47	37.64 ± 30.16	34.52 ± 28.77	40.54 ± 19.86	39.14 ± 20.17	104.29 ± 26.33*,§	169.47 ± 33.62*†,§	40.38 ± 27.52†,‡	36.24 ± 24.56	90.66 ± 31.63*,§	138.27 ± 22.43*,†,§	45.49 ± 20.48†,‡
TroponinT (ng/ml)	0.021 ± 0.007	0.018 ± 0.011	0.012 ± 0.017	0.008 ± 0.010	0.003 ± 0.003	0.245 ± 0.118*,§	0.854 ± 0.227*,†,§	0.268 ± 0.219*,‡,§	0.010 ± 0.005	0.112 ± 0.104*,§,¶	0.435 ± 0.305*,†,§,¶	0.196 ± 0.124*,‡,§

* Compared with BASE, *P* < 0.05.

† Compared with 2H, *P* < 0.05.

‡ Compared with 6H, *P* < 0.05.

§ Compared with sham-operation group, *P* < 0.05.

* compared with CME group, *P* <¶0.05.

2H: two hours after operation; 6H: six hours after operation; 1W: seven days after operation; TNF-α: Tumour Necrosis Factor-alpha; IL-6: interleukin-6.

### Myocardial apoptosis

**Figure 2:**
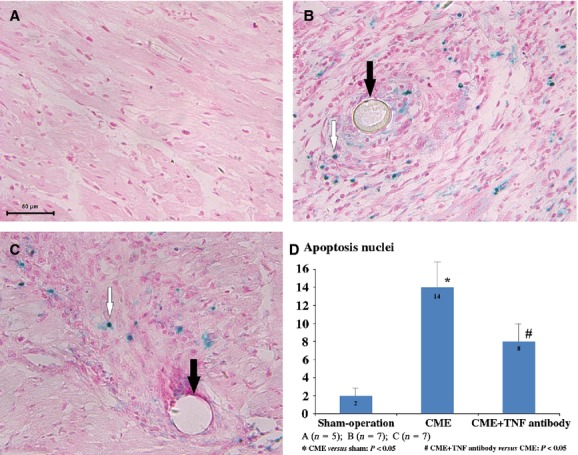
Cardiomyocyte apoptosis stained by TUNEL method in three groups (×400). (**A**) Sham-operation group; (**B**) CME group; (**C**) treatment group; (**D**) comparison of the average number of apoptosis nuclei among three groups. White arrows pointed to apoptosis nuclei with blue colour; black arrows pointed to microspheres.

**Figure 3:**
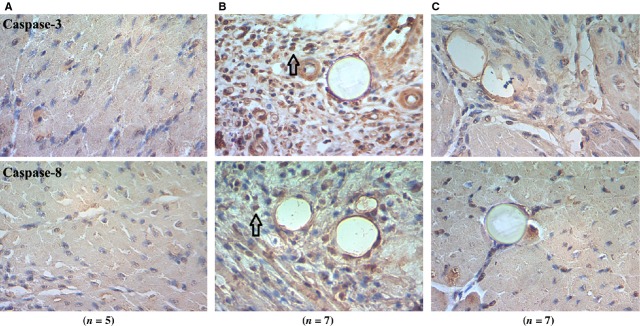
Protein expression of caspase-3 and caspase-8 were detected by immunohistochemistry (×400). (**A**) Sham-operation group; (**B**) CME group; (**C**) treatment group; Black arrows pointed to positive nuclei.

We performed TUNEL staining and found that most apoptotic cardiac cells were located in the infracted area. However, a few of these cells were also found around the infracted regions. The average number of apoptotic cardiomyocyte nuclei in the CME group was significantly higher than in the sham-operation group (14 ± 3 *versus* 2 ± 1, *P* < 0.01). Pre-treatment with TNF-α antibody significantly decreased apoptosis (8 ± 2 *versus* 14 ± 3, *P* < 0.05; Fig.2). Occurrence of cell apoptosis was also confirmed by immunohistochemistry of caspase-3 and caspase-8. We found that both of caspase-3 and caspase-8 were increased after CME and decreased by TNF-α antibody treatment (Fig.3).

**Figure 4:**
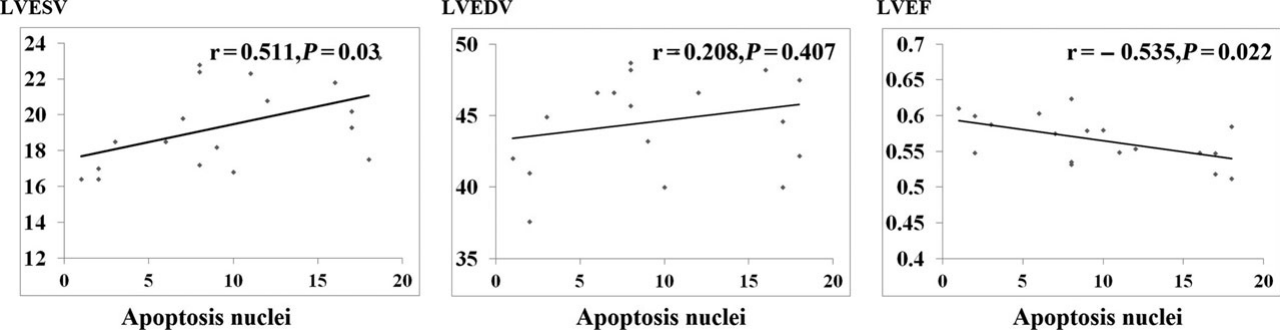
Correlations between the average number of cardiomyocyte apoptosis nuclei and cardiac function.

The correlation between apoptotic nuclei and cardiac function was analysed by Spearman test. It demonstrated that the average number of apoptotic cardiomyocytes positively correlated with LVESV (*r* = 0.511, *P* = 0.030) and negatively correlated with LVEF (*r* = −0.535, *P* = 0.022). There was no significant correlation with LVEDV (*r* = 0.208, *P* = 0.407; Fig.4).

### Protein and mRNA expression

**Figure 5:**
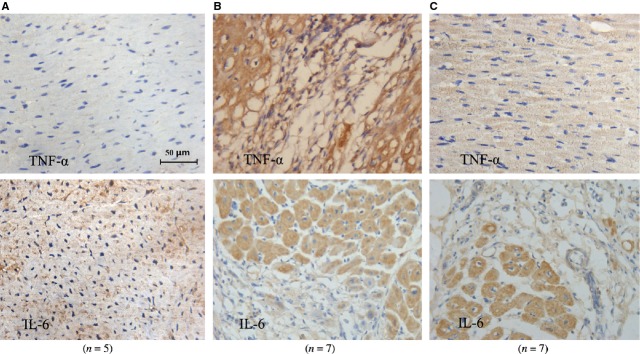
Protein expression of TNF-α and IL-6 were detected by immunohistochemistry (×400). (**A**) Sham-operation group; (**B**) CME group; (**C**) treatment group.

**Figure 6:**
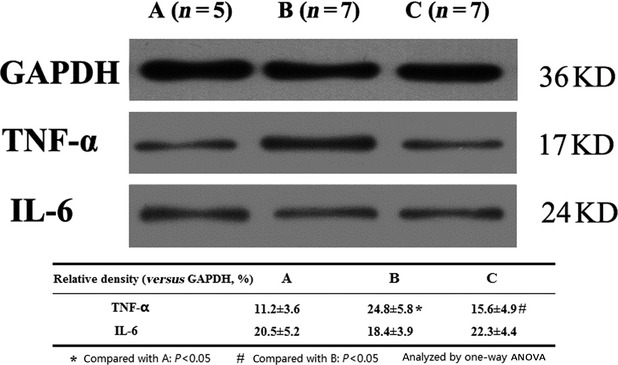
Protein expression of TNF-α and IL-6 were detected by Western blot. (**A**) Sham-operation group; (**B**) CME group; (**C**) treatment group.

Compared with the sham-operation group, protein expression of TNF-α detected by immunohistochemistry and Western blot was increased significantly in the CME group; in contrast, it was markedly decreased in the TNF-α antibody therapy group. However, there was no significant difference in IL-6 expression between the CME and TNF-α antibody treatment groups (Table5, Figs5 and 6).

**Table 5:** Positive-staining area per cent of immunohistochemistry among three groups

(%)	Sham-operation group (*n* = 5)	CME group (*n* = 7)	Antibody treatment group (*n* = 7)
TNF-α	2.6 ± 3.2	9.4 ± 5.7*	4.7 ± 6.2†
IL-6	1.8 ± 2.8	11.6 ± 4.4	10.2 ± 5.6

* Compared with sham-operation group, *P* < 0.05.

† Cmpared with CME group, *P* < 0.05.

TNF-α: tumour necrosis factor-alpha; IL-6: interleukin-6.

**Figure 7:**
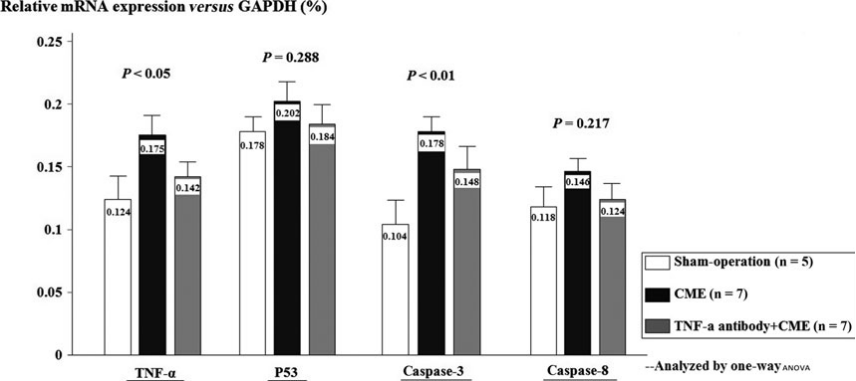
Quantitative analysis of TNF-α, P53, caspase-3 and caspase-8 mRNA expression.

mRNA expression of TNF-α, caspase-3 and caspase-8 was increased in the CME group 1 week after the procedure. TNF-α antibody treatment significantly suppressed mRNA expression of TNF-α, caspase-3 and caspase-8. However, P53 mRNA expression, which was one of important participants in the process of apoptosis, was unaltered by TNF-α antibody treatment (Fig.7).

### Collagen analysis

**Figure 8:**
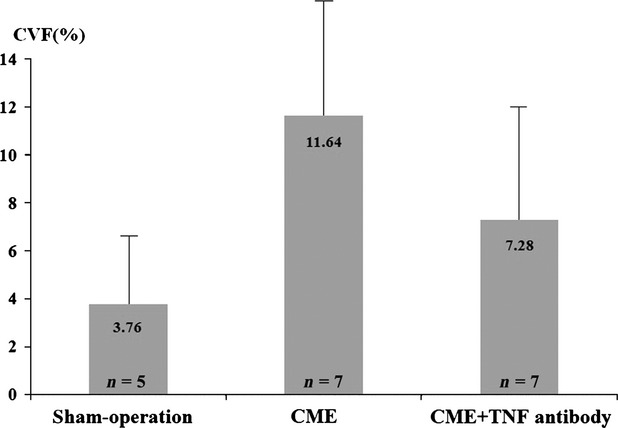
The average collagen volume fraction among three groups.

Masson Trichrome staining revealed that collagen volume was increased in the anterior myocardium in CME group 1 week after operation. The average CVF in CME group (11.64 ± 4.69%) was significantly higher than that in sham-operation group (3.76 ± 3.05%, *P* < 0.05). TNF-α antibody treatment greatly reduced CVF after CME (7.28 ± 4.46%, compared to the CME group, *P* < 0.05, Fig.8).

## Discussion

CME occurring secondary to spontaneous or therapeutic plaque rupture is one of major complications associated with acute coronary syndromes and coronary intervention 29,30. CME significantly influences the prognosis of coronary heart disease and coronary revascularization therapy. Atherosclerotic material and platelet thrombi dropping may occlude coronary microcirculation and result in microinfarction. A typical consequence of CME is contractile dysfunction 1,31. In this study, we not only confirmed cardiac dysfunction after CME in mini-pigs by MRI, but also found that the local collagen volume was elevated significantly (Fig.8), thus demonstrating the presence of cardiac dysfunction and remodelling by increasing interstitial substance after CME.

Contractile dysfunction caused by CME is mediated by the local inflammatory response and characterized by leucocyte infiltration, especially expression of TNF-α 7,8. Several experimental studies have supported that TNF-α is involved in the deterioration of heart function after CME 7,8,13. Skyschally *et al*. 12 found that pre-treatment with TNF-α antibody attenuated the progressive cardiac dysfunction following CME. This is likely because of the vast role of TNF-α in a number of different pathophysiological processes, including oxidative stress 20, nitric oxide synthase expression 21,22, activation of NF-κB 23, apoptosis 24 and so on. However, prior to this work, the relationship between TNF-α and cardiac apoptosis in CME had not been examined. Thus, we performed this study to clarify the role of TNF-α-induced apoptosis in heart dysfunction after CME and the effect of TNF-α antibody treatment.

Myocardial TNF-α expression at both the protein and mRNA level was decreased by treatment with TNF-α selective antagonist. Serum level of TNF-α was not significantly altered, maybe it was associated with less dosage (2 mg/kg) or method of administration (intracoronary, not intravenous) or compensatory mechanism. Adalimumab (Humira), which was intracoronary injection in our study, was one of recombinant human monoclonal anti-TNF-α antibodies. It could combine with TNF-α specially, and block the interaction between TNF-α and its receptors (p55 and p75). Its pharmacokinetics half-time was 12 to 14 days. We found that TNF-α antibody improved cardiac dysfunction after CME (Table3), which confirmed the involvement of TNF-α in CME-induced heart dysfunction. Previous studies have established a role for TNF-α in oxidative stress and oxidative modification 32, signal transduction leading to sphingosine production 8 and NF-κB activation 25 after CME. Cardiomyocyte apoptosis is a critical determinant of heart failure progression 33,34. However, it was still unclear the occurrence of cardiac apoptosis in CME and the association between TNF-α expression and cardiac apoptosis, especially in huge animals (such as pigs or dogs). In this experimental study, cardiomyocyte apoptosis was significantly increased in CME group. This TUNEL detecting kit applied in our study was specifically enhanced the labelling of apoptotic cardiac cells. We also found that the improvement of cardiac function was associated with the average number of apoptosis (Fig.4). These findings implied that cardiomyocyte apoptosis was partly involved in the progression of cardiac dysfunction, especially systolic function (Table3 and Fig.2). Importantly, rather than direct inhibition of cardiac apoptosis by special agent, TNF-α antibody treatment did decreased the process of cardiac apoptosis in our study, which also confirmed the link between TNF-α and cardiac apoptosis in CME.

Cardiomyocyte apoptosis has been documented as a critical form of cell death in ischaemia and reperfusion damage. A key component to apoptotic signalling pathways is the activation of caspases, a group of cysteinyl-aspartate-directed proteases. Caspases are typically activated by proteolytic cleavage 35. In turn, active caspases cleave vital substrates in the cell, leading to cellular death. In the heart, confirmed caspases substrates include crucial molecules of cellular homoeostasis such as α-actin, α-actinin, α/β-myosin heavy chain, tropomyosin and cardiac troponins 36. As a key regulatory component of apoptosis, the Bcl-2 family of proteins consists of death antagonists (Bcl-2) and death agonists (Bax), which function to protect or disrupt the integrity of the mitochondrial membrane and control the release of pro-apoptotic intermembrane proteins 37,38. However, few studies have examined apoptotic signal transduction in the heart after CME. Here, we find that expressions (mRNA and protein level) of caspase-3 and caspase-8 after CME were both enhanced significantly. Compared with those in CME group, expressions of caspase-3 and caspase-8 in TNF-α antibody therapy group were decreased, although there were no statistical different in serum TNF-α. These results demonstrated that apoptosis occurring after CME are likely mediated by TNF-α, which induced expression of both caspase-3 and caspase-8.

We should note some of our study's limitations. First, there were only a small number of mini-pigs investigated. Second, a prolonged experimental period should be warranted to see the long-term effects of TNF-α on cardiac apoptosis after CME. Third, multiple doses of TNF-α antibody should be taken into account. In addition, more *in vitro* research is required to clarify the detailed signal transduction involved cardiomyocyte apoptosis. Finally, microspheres are chemically inert and lack any chemoattractant properties; thus, they are quite different from microemboli identified in patients at autopsy or retrieved during PCI. In conclusion, further research should be performed taking these limitations into account.

## Conclusion

TNF-α-induced cardiomyocyte apoptosis is likely involved in cardiac dysfunction after CME. TNF-α antibody therapy suppresses cardiomyocyte apoptosis and improves early cardiac function after CME.
